# Prevalence of morbidities across the lifespan for adults with spinal muscular atrophy: a retrospective cohort study

**DOI:** 10.1186/s13023-023-02872-6

**Published:** 2023-08-31

**Authors:** Daniel G. Whitney, Erin E. Neil Knierbein, Alecia K. Daunter

**Affiliations:** 1 Department of Physical Medicine and Rehabilitation, University of Michigan, Ann Arbor, MI USA; 2 Institute for Healthcare Policy and Innovation, University of Michigan, Ann Arbor, MI USA; 3 Department of Pediatrics, University of Michigan, Ann Arbor, MI USA

**Keywords:** Adults, Spinal muscular atrophy, Morbidity, Clinical epidemiology

## Abstract

**Background:**

Recently approved treatments for spinal muscular atrophy (SMA) may shift clinical care priorities to secondary complications associated with SMA-related aging. To date, there is little knowledge about the natural history of morbidities across the adult lifespan for SMA. The objective of this study was to identify the prevalence and odds ratio (OR) of various morbidities among adults with vs. without SMA prior to SMA-related treatment.

**Methods:**

This was a retrospective cohort study that accessed Medicare fee-for-service and commercial claims data from 01/01/2008-12/22/2016. Data from adults ≥ 18 years old with SMA and without SMA matched (1:200 case:control) on demographics, region, and study entry year were included. The prevalence of 30 morbidities across physiologic systems (e.g., cardiovascular, metabolic, musculoskeletal, urinary) and mental health disorders was examined. Age- and sex-adjusted OR was estimated using logistic regression for each morbidity and effect modification by age and sex was tested.

**Results:**

There were 2,427 adults with SMA (mean [SD] age, 59.7 [17.4] years; 49.0% female) and 484,528 matched adults without SMA. Adults with vs. without SMA had a higher prevalence and adjusted OR of all 30 morbidities, ranging from OR = 1.61 (95% CI = 1.45–1.80) for hypothyroidism to OR = 7.80 (95% CI = 7.10–8.57) for fluid/electrolyte disorders. There was effect modification by age for 24 morbidities. The OR was highest for the youngest age group (18–40 years; OR range, 2.38 to 117.7; all *P* < 0.05) and declined with older age groups, but still remained significantly elevated in the oldest age group (≥ 75 years; OR range, 1.30 to 5.96; all *P* < 0.05).

**Conclusions:**

The limitations of this study are that evidence of morbidities were limited to diagnostic claims and information on SMA type and symptoms or onset were not available. In conclusion, adults with SMA had a higher and earlier prevalence of a variety of morbidities across physiological systems and mental health disorders.

**Supplementary Information:**

The online version contains supplementary material available at 10.1186/s13023-023-02872-6.

## Background

Spinal muscular atrophy (SMA) is a rare neuromuscular condition characterized by a group of hereditary, progressive motor neuron destructive diseases [1]. SMA encompasses varied severities, which is often clinically classified based on the timing of onset of motor dysfunction. For example, SMA type I is the most common form (approximately half of SMA patients). Hypotonia, delayed motor function milestones, and other motor neuron related issues (e.g., feeding, respiration) present within the first 6-months of life for individuals with SMA type I and the natural age of death is < 2 years old [2]. SMA type II (~ 30% of SMA patients) and type III (~ 10% of SMA patients) are less severe forms, present as never acquiring the ability to walk (type II) or losing the ability to walk in childhood (type III). The natural age of death for individuals with SMA type II and III is > 2 years old and adulthood, respectively [2, 3]. SMA type IV is the least common (~ 5% of SMA patients) and the onset of functional decline begins in the adult years [4, 5].

What links all individuals with SMA is some degree of motor dysfunction in the lifespan, which can increase the risk for a variety of morbidities across physiological systems, especially with aging. Moreover, the disease severity of SMA correlates with the functional protein levels of the survival motor neuron protein [6, 7]. This protein can be expressed in tissue outside of the spinal cord, suggesting the possibility for a direct pathological role of SMA on non-neuromuscular physiologic systems [8]. To date, there is little research examining the onset of morbidities across the adult lifespan for individuals with SMA [9, 10]. This is mainly because the majority of SMA-related research has focused on elucidating the molecular mechanisms of SMA and developing groundbreaking disease-modifying therapies. Beginning in December 2016, several therapies for SMA have been approved by the U.S. Food and Drug Administration. These therapies have shown efficacy in reducing mortality and the need for mechanical ventilation and improving motor function [5, 11–17]. However, there may still be deficits in motor abilities, especially if therapy is initiated after the disease has become symptomatic. Thus, identifying morbidity profiles across physiologic systems as individuals with SMA age into and throughout their adult years is necessary to assist in prioritizing clinical care to manage the person with SMA as opposed to the physiologic system SMA primary impacts.

The primary objective of this study was to identify the prevalence and odds ratio (OR) of morbidities across several physiologic systems for adults with SMA as compared to the general population of adults without SMA. This study used real-world clinical data in the “pre-treatment era” prior to the first approved disease-modifying therapy to establish a “baseline” of morbidity profiles for adults with SMA. It was hypothesized that adults with SMA would have a higher prevalence and OR of all examined morbidities, and that the prevalence and OR of many morbidities would be elevated earlier in the adult lifespan compared to adults without SMA.

## Methods

### Data sources

This was a retrospective cohort study that accessed administrative claims from the Medicare fee-for-service (20% random sample) database Part A and B (hospital and medical insurance) and Optum’s de-identified Clinformatics® Data Mart Database. This study did not access Medicare’s Part C or D (Medicare Advantage Plan and prescription drug coverage). Patient-level claims from 01/01/2008-12/22/2016 were ascertained to examine outcomes prior to the first U.S. Food and Drug Administration approved disease-modifying therapy for SMA. Medicare is a federal program that provides medical insurance to individuals ≥ 65 years old with or without disabilities and with certain disabilities if younger than 65 years old, including SMA. Optum is a national database of individuals with commercial medical insurance or with a Medicare Advantage Plan. The cohort with SMA was obtained from the Medicare and Optum database. As Optum provides greater representation of adults < 65 years old compared to Medicare and reasonable representation of adults ≥ 65 years old, the cohort without SMA was obtained from the Optum database to provide a representative background population across the adult lifespan.

Administrative claims are used for billing reimbursement of healthcare services. Researchers can identify medical conditions, via clinician-based diagnoses, by searching patient-level claims for unique codes attached to the claim. All medical conditions examined in this study were identified by International Classification of Diseases, Ninth or Tenth Revision, Clinical Modification (ICD-9/ICD-10) codes, which are listed in **Additional file 1**. The University’s Institutional Review Board approved this study as non-regulated as the data are de-identified prior to administering to researchers.

### Cohort selection

**Fig. 1:**
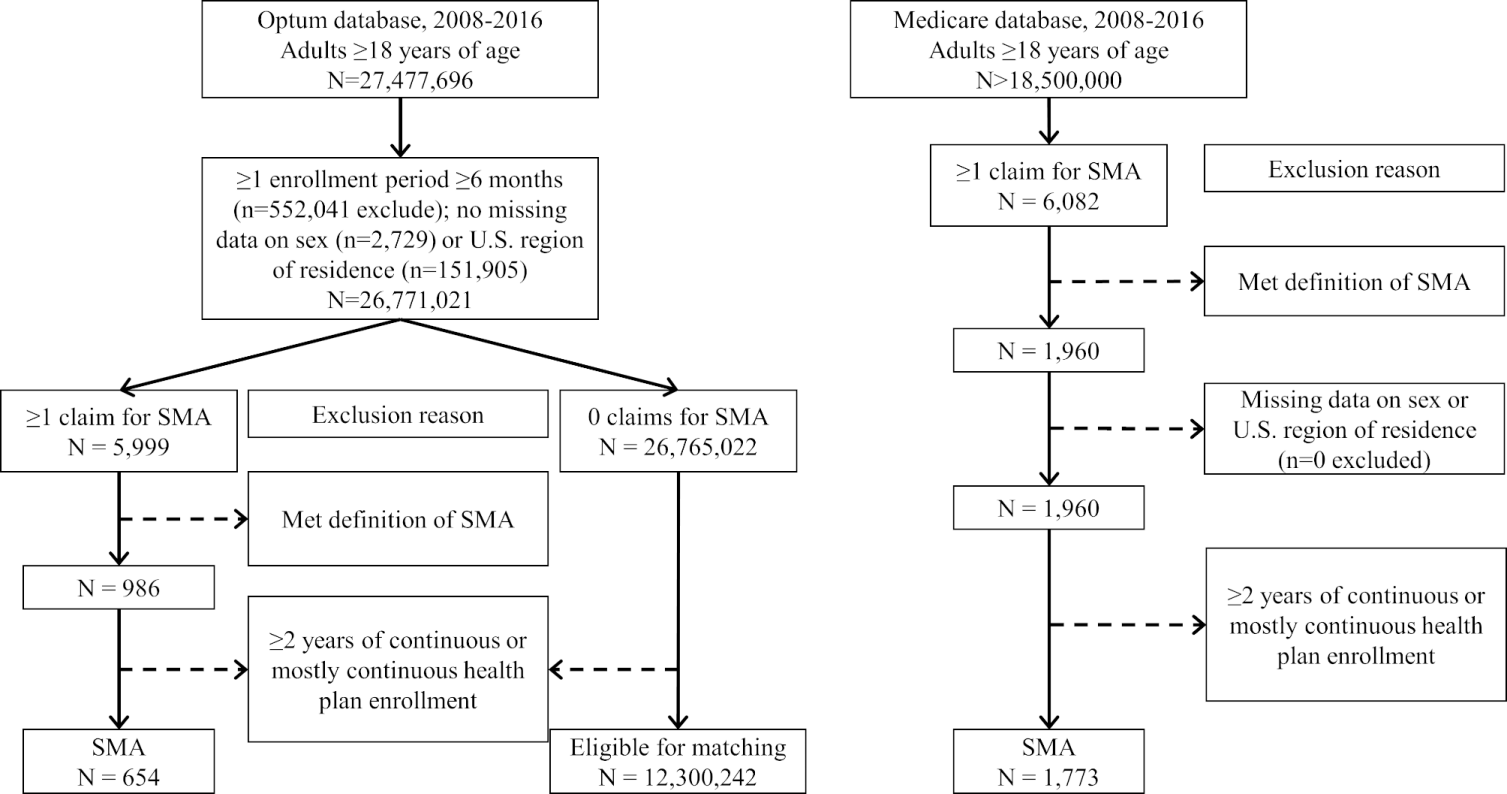
Flow chart. Flow chart of inclusion/exclusion criteria from the Optum database to obtain adults with and without spinal muscular atrophy (SMA) and from the Medicare database to obtain adults with SMA

A flow chart of inclusion/exclusion steps is presented in Fig. 1. Adults ≥ 18 years old with SMA were identified in the Medicare database as ≥ 1 inpatient claim for SMA or ≥ 2 outpatient claims for SMA, where each outpatient claim was on a separate day within 12-months of one another. This is consistent with recommendations by the Chronic Conditions Data Warehouse. For Optum, diagnoses were not separated by inpatient or outpatient services due to differences in the underlying data structure. Therefore, the algorithm of ≥ 2 claims for SMA described above was used regardless of where the claim originated.

To be included in the analysis, adults had ≥ 2-years of continuous or mostly continuous enrollment in a health plan between 01/01/2008-12/22/2016. It is not uncommon for insurance enrollment periods to have a short break for reasons unrelated to the outcomes and conclusions drawn from a study like this. Therefore, this study allowed for up to 3 breaks in enrollment, where each break was < 30 days and separated by ≥ 6-months of continuous enrollment. This approach helps to balance detection and selection biases to draw meaningful conclusions regarding morbidity prevalence across a 2-year period. The majority of the cohort with SMA in this study had an unbroken 2-year enrollment (93.7%), while 5.8% had 1 break, 0.5% had 2 breaks, and 0% had 3 breaks in enrollment.

The start and end of the 2-year period was centered around the first SMA claim date as much as possible. This method was employed as the diagnostic codes for SMA do not distinguish between the types of SMA. Other research teams have used the age at the first diagnostic claim for SMA, but this was in pediatrics [18] and the claims data available in this study do not include information from birth. Although, we anticipate that the majority of the cohort will have pediatric-onset SMA (types I-III) as opposed to adult-onset SMA (type IV), given that the latter contributes to ~ 5% of the population with SMA [5]. For those with > 1-year of enrollment prior to and after the first SMA claim date, the start of the 2-year period (to examine morbidity prevalence) was 1-year prior to the first SMA claim date and the end date was 2-years after the start date. For those with < 1-year of enrollment prior to their first SMA claim date, the first enrollment date was the start date and the end date was 2-year after. For those with < 1-year of enrollment after the first SMA claim date, the end date was the last available date and the start date was 2-years prior.

Adults with 0 claims for SMA were considered for inclusion in the cohort without SMA, and their start and end dates were randomly assigned within 01/01/2008-12/22/2016. To enhance interpretation of crude prevalence estimates, adults without SMA were matched (without replacement) to each adult with SMA at a 1:200 ratio (case:control) for age (± 2 years), sex, U.S. region of residence, race (as white vs. non-white due to data limitations), and study entry year. The rationale for the 1:200 ratio was to enhance generalizability of the estimates from the comparison cohort. Since SMA is a rare population, smaller matching ratios commonly used in epidemiologic studies (e.g., 1:1, 1:5, 1:10) would result in a smaller comparison cohort. This can decrease generalizability of estimates derived from the non-SMA comparison cohort that was meant to reflect the general population without SMA.

### Morbidities

Selection of morbidities was guided by established and validated comorbidity indices using claims, including the Charlson [19, 20], Elixhauser [21], and Whitney [22, 23] Comorbidity Index. The Whitney Comorbidity Index was developed for adults with cerebral palsy and is more relevant to capture morbidity and multi-morbidity profiles for populations with neurological conditions than other commonly used indices. Collectively, there were 30 morbidities examined across physiological systems, such as cardiovascular, metabolic, respiratory, musculoskeletal, gastrointestinal, liver, kidney, and cancer, as well as mental health disorders. A multi-morbidity score was calculated as the sum of the 30 morbidities, which provides some form of evidence of medical complexity in terms of the number of physiologic systems impacted by morbidities.

For consistency across databases, the presence of each morbidity was defined by ≥ 2 claims (inpatient or outpatient, any position), where each claim was on a separate day within the 2-year period. The sensitivity and specificity of the examined morbidities ranges from fair to excellent. It is assumed that the ability to detect each morbidity does not differ by cohort as there is no evidence to suggest differential detection. Therefore, the relative effect estimate of each morbidity comparing adults with vs. without SMA is assumed to be more representative of the true relative difference.

### Characteristics

Age determined based on the study start date, sex, race, and U.S. region of residence were retrieved. To further characterize the Medicare cohort with SMA, the original reason for Medicare entitlement was obtained. Given the sample size, age was categorized as 18–39, 40–54, 55–64, 65–74, and ≥ 75 years old to capture various stages of the adult lifespan.

### Statistical analysis

Baseline descriptive characteristics were described for the cohorts. The prevalence of each morbidity was described by sex for adults ≥ 18 years old. The prevalence of each morbidity was also described by age group combining females and males. There were too few outcome cases to examine prevalence estimates by sex and age group simultaneously.

Logistic regression estimated the OR with 95% confidence intervals (CI) of each morbidity comparing adults (females and males combined) with vs. without SMA after adjusting for age (continuous) and sex. Effect modification by age and sex was tested for each comorbidity by including the main effects of cohort (SMA vs. non-SMA), age, and sex and their 2-way interactions. If there was evidence of effect modification (interaction term, *P* < 0.05) then additional analyses were performed that stratified by the variable. A 3-way interaction (cohort*age*sex) was not considered due to sample size limitations.

To compare the multi-morbidity score between cohorts, a generalized linear model with zero-inflated Poisson distribution was developed after adjusting for age (continuous) and sex. This approach utilizes a two-part model that is useful for dealing with overdispersion due to excess zero values common to multi-morbidity scores [24]. The first part of the model estimates the OR of having a zero vs. non-zero (i.e., ≥ 1) value, where an OR < 1.00 would indicate that the cohort with vs. without SMA is less likely to have 0 morbidities, and thus more likely to have ≥ 1 morbidity. The second part of the model estimates the incidence rate ratio (IRR) as the count of morbidities conditional on non-zero values; i.e., comparing SMA vs. without SMA that have ≥ 1 morbidity. Effect modification by age and sex was examined as described above.

Any variable where estimates had n ≤ 11 cases were suppressed as part of the Data Use Agreement for both databases to preserve patient de-identification. Analyses were performed using SAS version 9.4 (SAS Institute, Cary, NC, USA).

### Role of the funding source

This work was not supported by any funding source.

## Results

Baseline descriptive characteristics of adults with SMA (n = 2,427) and matched adults without SMA (n = 484,528) is presented in Table 1. The majority (99.5%) of the cohort with SMA had suitable matches at a ratio of 1:200, while the remaining 0.5% (n = 12) had suitable matches ranging from 25 to 197. These 12 adults with SMA and their corresponding matches were included in the analysis and no weighting was applied.

**Table 1:** Descriptive characteristics of adults with spinal muscular atrophy (SMA) and matched adults without SMA (w/o SMA)

	SMA (n = 2,427)	w/o SMA (n = 484,528)
Age, mean (SD)	59.7 (17.4)	59.5 (17.2)
18–39 years, % (n)	15.4 (374)	15.4 (74,513)
40–54 years, % (n)	19.3 (469)	18.9 (91,372)
55–64 years, % (n)	17.6 (428)	16.4 (79,462)
65–74 years, % (n)	27.8 (674)	29.1 (141,200)
≥75 years, % (n)	19.9 (482)	20.2 (97,981)
Sex, % (n)		
Female	49.0 (1,190)	49.0 (237,630)
Male	51.0 (1,237)	51.0 (246,898)
Race, % (n)		
Asian	1.8 (44)	2.5 (11,944)
Black	10.7 (259)	5.9 (28,423)
Hispanic	3.8 (92)	6.2 (29,814)
White	76.8 (1,864)	76.8 (372,119)
Other/unknown	6.9 (168)	8.7 (42,228)
U.S. region of residence, % (n)		
West	20.7 (501)	20.8 (100,542)
Midwest	21.4 (518)	21.5 (103,985)
South	39.6 (958)	39.6 (191,979)
Northeast	18.3 (442)	18.2 (88,022)
Insurance database, % (n)		
Commercial	27.0 (654)	100 (484,528)
Medicare fee-for-service	73.0 (1,773)	-
Original reason for Medicare entitlement		
Old age and survivor’s insurance	47.8 (848)	-
Disability insurance benefits (DIB)	51.0 (904)	-
End-stage renal disease (ESRD) or DIB + ESRD	1.2 (21)	-

SD, standard deviation

### Morbidities

**Fig. 2:**
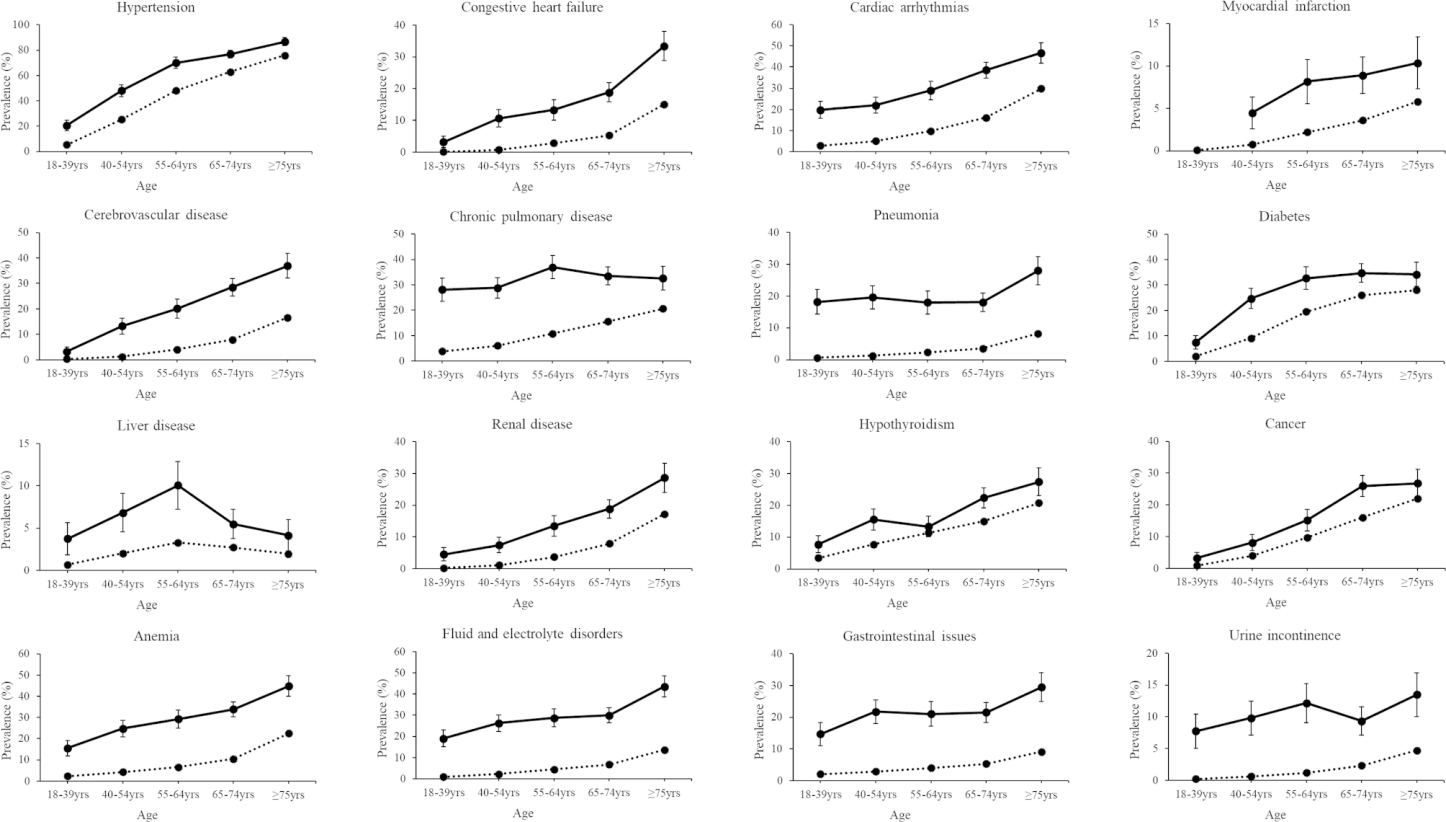
Prevalence of morbidities by age group part 1. Crude prevalence with 95% confidence intervals (vertical lines) for morbidities of the cardiovascular, respiratory, metabolic, digestive, and urinary systems, as well as anemia, fluid and electrolyte disorders, and gastrointestinal issues for adults (females and male combined) with (solid line) and without (dotted line) spinal muscular atrophy. Estimates are not reported if the number of outcome cases was less than 11 for patient de-identification purposes. For each prevalence estimate, the difference is statistically significant at *P* ≤ 0.05 if the 95% confidence interval does not overlap between cohorts

**Fig. 3:**
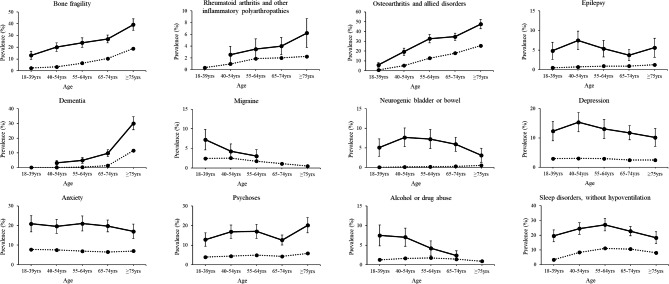
Prevalence of morbidities by age group part 2. Crude prevalence with 95% confidence intervals (vertical lines) for morbidities of the musculoskeletal and neurological systems, as well as mental health, substance abuse, and sleep disorders for adults (females and male combined) with (solid line) and without (dotted line) spinal muscular atrophy. Estimates are not reported if the number of outcome cases was less than 11 for patient de-identification purposes. For each prevalence estimate, the difference is statistically significant at *P* ≤ 0.05 if the 95% confidence interval does not overlap between cohorts

The sex-stratified prevalence of each of the 30 morbidities is presented in Table 2. The age group-stratified prevalence (females and males combined) of each morbidity is presented in Figs. 2 and 3. Adults with SMA had a significantly elevated age- and sex-adjusted OR for each morbidity compared to adults without SMA, which ranged from 1.61 (hypothyroidism) to 24.3 (intellectual disabilities) (Table 2).

**Table 2:** Sex-stratified prevalence and adjusted* odds ratio (OR) of morbidities for adults with spinal muscular atrophy (SMA) and matched adults without SMA (w/o SMA).

	SMA		w/o SMA		SMA vs. w/o SMA
	Female	Male	Female	Male	Females and males combined
	% (n)	% (n)	% (n)	% (n)	OR (95% CI)
Hypertension	61.8 (735)	64.9 (803)	46.2 (109,756)	48 (118,579)	2.45 (2.22, 2.70)
Chronic pulmonary disease	35.0 (416)	29.5 (365)	13.3 (31,541)	11.2 (27,616)	3.63 (3.32, 3.97)
Bone fragility	34.0 (405)	17.1 (211)	13.7 (32,474)	4.2 (10,403)	4.10 (3.71, 4.54)
Osteoarthritis	33.4 (398)	25.7 (318)	16.3 (38,686)	10.9 (26,928)	2.97 (2.70, 3.26)
Anemia	32.2 (383)	29.1 (360)	11.3 (26,921)	8.3 (20,580)	4.63 (4.22, 5.09)
Cardiac arrhythmias	31.6 (376)	33.1 (410)	13.3 (31,691)	14.2 (35,082)	3.38 (3.09, 3.71)
Fluid/electrolyte disorders	30.5 (363)	29.6 (366)	7.0 (16,599)	5.1 (12,714)	7.80 (7.10, 8.57)
Diabetes	25.3 (301)	30.9 (382)	16.6 (39,525)	20.2 (49,880)	1.79 (1.63, 1.97)
Hypothyroidism	25.0 (298)	11.6 (144)	18.3 (43,507)	6.8 (16,765)	1.61 (1.45, 1.80)
Gastrointestinal issues	24.1 (287)	20.0 (247)	6.3 (14,917)	3.7 (9,084)	5.69 (5.15, 6.28)
Cerebrovascular disease	22.9 (272)	20.9 (258)	6.5 (15,425)	6.7 (16,442)	4.59 (4.13, 5.11)
Anxiety	22.3 (265)	17.0 (210)	9.5 (22,666)	4.7 (11,552)	3.26 (2.95, 3.61)
Sleep disorders (excludes sleep-related hypoventilation)	22.3 (265)	22.6 (280)	7.6 (18,137)	9.5 (23,464)	3.11 (2.82, 3.42)
Pneumonia	19.6 (233)	21.1 (261)	3.4 (8,069)	3.4 (8,418)	8.00 (7.20, 8.89)
Congestive heart failure	17.8 (212)	15.8 (195)	5.0 (11,906)	5.5 (13,475)	4.18 (3.71, 4.70)
Psychoses	16.7 (199)	14.8 (183)	5.9 (14,041)	3.4 (8,478)	3.87 (3.46, 4.32)
Cancer	14.7 (175)	19.7 (244)	10.4 (24,711)	12.7 (31,451)	1.63 (1.46, 1.82)
Depression	14.7 (175)	10.3 (127)	3.7 (8,798)	1.8 (4,400)	5.15 (4.56, 5.83)
Renal disease	14.3 (170)	16.6 (205)	6.3 (14,915)	7.0 (17,355)	2.77 (2.45, 3.12)
Urine incontinence	11.3 (135)	9.7 (120)	2.7 (6,418)	1.3 (3,177)	6.13 (5.35, 7.03)
Dementia	11.2 (133)	9.5 (118)	3.7 (8,760)	1.9 (4,741)	5.11 (4.36, 5.99)
Liver disease	6.5 (77)	5.6 (69)	2.1 (4,975)	2.3 (5,760)	2.82 (2.38, 3.34)
Myocardial infarction	5.6 (67)	8.3 (103)	1.9 (4,438)	3.6 (8,785)	2.72 (2.32, 3.20)
Migraine	5.4 (64)	1.0 (12)	2.5 (5,873)	0.7 (1,627)	2.08 (1.65, 2.63)
Neurogenic bladder or bowel	5.2 (62)	6.4 (79)	0.2 (507)	0.3 (764)	23.6 (19.3, 28.3)
Rheumatoid arthritis and other inflammatory polyarthropathies	5.1 (61)	2.2 (27)	2.2 (5,162)	1.0 (2,497)	2.34 (1.89, 2.90)
Epilepsy	5.0 (60)	5.5 (68)	0.9 (2,140)	0.9 (2,115)	6.28 (5.24, 7.52)
Alcohol or drug abuse	4.0 (48)	4.4 (55)	1.0 (2,350)	1.8 (4,401)	3.15 (2.58, 3.84)
Intellectual disabilities	1.8 (22)	1.9 (23)	0.1 (155)	0.1 (224)	24.3 (17.8, 33.2)
Sleep-related hypoventilation	1.0 (12)	< 1%**	0.1 (127)	0.1 (161)	11.8 (7.2, 19.2)

CI, confidence interval. *Adjusted for age (continuous) and sex. **Data suppressed as N ≤ 11 for patient de-identification purposes

There was strong evidence of effect modification by age for 24 morbidities (*P* for interaction, all ≤ 0.006). In general, the OR comparing adults with vs. without SMA was the highest for the youngest age group (18–39 year olds) and declined with older age groups. However, the OR of all of these morbidities in the oldest age group (i.e., ≥ 75 year olds) was still significantly elevated, ranging from 1.30 (cancer, 95% CI = 1.06–1.59) to 5.96 (neurogenic bowel/bladder, 95% CI = 3.54-10.0) (**Additional file 2**).

There was strong evidence of effect modification by sex for 13 morbidities (*P* for interaction, < 0.001 to 0.038). The OR for these morbidities was significantly elevated for females and males with vs. without SMA, but the strength of the association differed by sex. In general, the OR for females (with vs. without SMA) was higher than males (with vs. without SMA) for the cardiovascular diseases and sleep disorders, while the OR for males (with vs. without SMA) was higher than females (with vs. without SMA) for bone fragility, dementia, mental health disorders, and nutrition/gastrointestinal disorders (Table 3).

**Table 3:** Odds ratio (OR) of morbidities with effect modification by sex, comparing adults with spinal muscular atrophy (n = 2,427) to matched adults without spinal muscular atrophy (n = 484,528)

	Females	Males
	OR (95% CI)	OR (95% CI)
Congestive heart failure	4.81 (4.07, 5.69)	3.66 (3.09, 4.32)
Cerebrovascular disease	5.04 (4.33, 5.87)	4.20 (3.62, 4.89)
Bone fragility	3.65 (3.20, 4.15)	4.91 (4.21, 5.74)
Dementia	3.94 (3.17, 4.91)	6.94 (5.54, 8.70)
Depression	4.49 (3.82, 5.28)	6.32 (5.25, 7.61)
Anxiety	2.72 (2.37, 3.12)	4.17 (3.59, 4.84)
Psychoses	3.20 (2.74, 3.73)	4.88 (4.17, 5.72)
Alcohol or drug abuse	4.22 (3.15, 5.64)	2.57 (1.96, 3.37)
Sleep disorders (excludes sleep-related hypoventilation)	3.49 (3.04, 4.01)	2.80 (2.45, 3.20)
Sleep-related hypoventilation	18.9 (10.4, 34.2)	*
Fluid/electrolyte disorders	6.84 (5.97, 7.82)	8.89 (7.80, 10.1)
Gastrointestinal issues	4.94 (4.30, 5.66)	6.72 (5.83, 7.76)
Urine incontinence	4.78 (3.96, 5.77)	8.55 (7.02, 10.4)

CI, confidence interval. *Too few outcome events to provide a meaningful estimate

### Multi-morbidity

The median (interquartile range) number of morbidities was 5 [2, 8] for females with SMA, 2 (0, 4) for females without SMA, 4 [2, 8] for males with SMA, and 1 (0, 3) for males without SMA. The number of morbidities in the following analysis were examined from 0 to ≥ 15, as the number beyond 15 morbidities became exceedingly small.

There was evidence of effect modification by sex in the zero-inflation model (*P* for interaction, 0.018), but not in the count model (*P* for interaction, 0.530). There was evidence of effect modification by age for females and males in the zero-inflated model (*P* for interaction, 0.015 and 0.010, respectively) and count model (*P* for interaction, both < 0.001). The results are presented in Table 4 by sex for the full cohort and after stratifying by age group. Females and males with vs. without SMA had a lower OR of zero-values in the zero-inflation model, indicating a higher proportion with ≥ 1 morbidity. The strength of the association declined with older age. Females and males with vs. without SMA had a higher IRR in the count model, indicating that among those with ≥ 1 morbidity, adults with SMA had on average a higher number of morbidities per person. The strength of the association declined with older age, but still remained significantly elevated across all age groups.

**Table 4:** Results for the main effect (full cohort) and effect modification (interaction) from the zero-inflated Poisson model for any morbidity (yes/no, zero-inflation model) and number of morbidities (count model) for adults with spinal muscular atrophy (SMA) compared to matched adults without SMA (w/o SMA).

	Zero-inflated model	Count model
	OR (95% CI)	IRR (95% CI)
Full cohort*		
Females		
SMA vs. w/o SMA	0.26 (0.20, 0.33)	2.11 (2.06, 2.16)
Males		
SMA vs. w/o SMA	0.16 (0.12, 0.22)	2.15 (2.10, 2.21)
**Age interaction**		
Females		
SMA vs. w/o SMA		
18–39 years	0.23 (0.16, 0.33)	3.33 (3.07, 3.60)
40–54 years	0.34 (0.23, 0.50)	3.00 (2.82, 3.20)
55–64 years	0.14 (0.06, 0.34)	2.24 (2.10, 2.38)
65–74 years	0.71 (0.46, 1.09)	2.00 (1.91, 2.09)
≥75 years	**	1.75 (1.67, 1.83)
Males		
SMA vs. w/o SMA		
18–39 years	0.14 (0.08, 0.22)	2.91 (2.61, 3.24)
40–54 years	0.20 (0.13, 0.30)	2.83 (2.66, 3.02)
55–64 years	0.23 (0.13, 0.41)	2.35 (2.23, 2.49)
65–74 years	0.37 (0.22, 0.61)	2.13 (2.04, 2.22)
≥75 years	**	1.72 (1.63, 1.81)

OR, odds ratio; IRR, incidence rate ratio; CI, confidence interval. Age (continuous) was a covariate in the full cohort model. *Estimates are adjusted by age (continuous). **Too few with 0 morbidities to provide reasonable estimates. The zero-inflation model is a logistic regression model, predicting whether the individual had zero vs. non-zero (i.e.¸≥1) morbidities. An OR < 1.00 indicates a lower likelihood of having zero morbidities. The count model is a Poisson regression model, predicting the number of morbidities among those with non-zero values

## Discussion

The findings from this study suggest that adults with vs. without SMA had a higher prevalence and OR of morbidities across several physiologic systems and multi-morbidity that were disproportionately occurring at younger ages. This is important as many of the examined morbidities are typically associated with advanced aging (e.g., ≥ 65 years old) and may be overlooked clinically for young and middle-aged adults with SMA, thus missing out on critical opportunities for early detection and prevention. These findings highlight the need for clinical awareness of a high-early burden of morbidities as children with SMA age into and throughout their adult years, such as cardiorespiratory diseases, diabetes, renal disease, bone fragility, and mental health disorders.

The heterogeneity of physiologic systems impacted by morbidities observed in this study is consistent with prior studies. For example, it is known that bone fragility, including osteoporosis and fractures, and respiratory complications are more common among individuals with vs. without SMA [25–27]. A study by Lipkin et al. [9] reported a higher prevalence of morbidities across cardiovascular, metabolic, skeletal, gastrointestinal, and reproductive systems among individuals ≤ 65 years old with vs. without SMA. Similarly, Mouchet et al. [10] reported an elevated prevalence of non-neurological manifestations among individuals ≤ 65 years old with vs. without SMA, including endocrine, metabolic, skeletal, and circulatory systems. This study complements this prior literature in that the prevalence of morbidities were stratified by age and sex and extended > 65 years of age to provide a more detailed assessment of morbidities across the lifespan.

In the current study, the most common morbidities that were present in at least 25% of females with SMA were hypertension, chronic pulmonary disease, bone fragility, osteoarthritis, anemia, cardiac arrhythmias, fluid/electrolyte disorders, diabetes, and hypothyroidism. For males with SMA, it was hypertension, cardiac arrhythmias, diabetes, fluid/electrolyte disorders, chronic pulmonary disease, anemia, and osteoarthritis. The adjusted OR suggests that the morbidities in at least 25% of adults with SMA were 1.61 (hypothyroidism) to 7.80 (fluid/electrolyte disorders) compared to matched adults without SMA. This highlights the magnitude of the problem given the large OR based on morbidities that have a high absolute prevalence in adults with SMA (≥ 25%). It is noteworthy to mention the elevated prevalence and adjusted OR of other debilitating morbidities, including renal disease, dementia, and mental health disorders.

Claims data provides evidence about the presence of a morbidity. Claims data does not inform on the severity of the morbidity and this study did not examine if and how each morbidity was being managed through clinical interventions or its impact on quality of life. Thus, this study provides a numerical approximation regarding the potential extent of (multi-)morbidity prevalence for adults with SMA, but not related qualitative aspects. Further, due to the range of sensitivity/specificity of identifying morbidities using claims, the absolute prevalence of morbidities observed in this study may vary with the true absolute prevalence observed in the greater population. In the absence of evidence, an assumption was made that there is negligible differential detection of morbidities by claims across cohorts. On one hand, individuals with SMA are more likely to use healthcare services [28] which increases the chances for a diagnosis. On the other hand, premature morbidity onset can be overlooked clinically for many conditions that do not cause pain or are visually obvious. Taken together, speculation follows that the extent of differential detection bias by cohort is unlikely to be large enough to overturn the broad conclusions drawn regarding a generally higher and earlier prevalence and OR of morbidities across physiologic systems for adults with vs. without SMA.

A strength of this study was the reasonably large sample of adults, considering that SMA is a rare condition and little research has been conducted for this adult population. Sequestering a sufficient volume of health-related data to provide clinically meaningful information from rare clinical populations is a grand challenge, and little is known about morbidity outcomes as people with SMA transition into and throughout adulthood. Another strength was the number of morbidities investigated. The comprehensive assessment of clinically diagnosed morbidities across physiologic systems and mental health disorders among adults with SMA may prompt for improved screening strategies and identification of at-risk individuals.

There are limitations to this study in addition to what was mentioned above. First, claims data does not reliability differentiate by SMA type, there is no genetic information to phenotype SMA, and there was no reliably available proxy information for adults for this study, such as age when SMA-related symptoms first emerged. The survival rate differs as a function of the type of SMA [26]. Thus, the elevated prevalence of morbidities in the younger adult cohort may reflect a more medically complex sector of the SMA population (e.g., proportionally more SMA type I-III). However, even in the older age groups, which would presumably be relatively less medically complex patients with SMA (e.g., proportionally more SMA type IV), the morbidity prevalence was still substantially elevated. Future research is needed to distinguish age-related morbidity profiles by SMA type to further assist clinical care in identification of at-risk patients.

Second, the validity of using claims to identify people with SMA is unknown. Prior validation studies have found that identification of people with motor neuron disease to have a sensitivity of ~ 80–93% and a specificity ≥ 99.0%, but the codes used encompassed rather than isolated SMA [29]. The algorithm used in this study is consistent with the Chronic Conditions Data Warehouse for neuromuscular and muscular dystrophy populations and balances sensitivity and specificity. Although, other studies have used slightly different algorithms to identify SMA, such as ≥ 1 inpatient claim or ≥ 2 outpatient claims within 30 days of one another, or ≥ 1 inpatient claim or ≥ 3 outpatient claims [9, 30]. Nevertheless, selection bias is possible, such that this study included patients with SMA who received more clinical care, which could overestimate the extent of morbidity prevalence for the greater adult population with SMA.

Third, generalizability of study findings for the greater adult population with SMA is not exactly known. While studies of prevalence and incidence for this rare population have significant challenges [31], reported prevalence estimates range from 1 to ~ 7 per 100,000 people; although, these studies were among those < 20 years old [31–35]. In the current study, there were 654 adults with SMA identified from the Optum database and 12,300,242 adults without SMA from the Optum database that met all other eligibility criteria prior to matching. The prevalence of SMA in this Optum cohort was 5.3 per 100,000 adults, which is within the range previously reported [31–35]. A prevalence estimate from the Medicare database was not possible due to differences in enrollment not allowing for a full age spectrum background population for those without SMA. Further, to be eligible for analysis, this study required 2-years of continuous enrollment. Given the elevated mortality rate in SMA, the cohort with SMA included in this analysis may be healthier than the population with SMA in general. The difference in overall health is likely less pronounced for the cohort without SMA included in this analysis compared to the general population without SMA.

## Conclusion

Adults with SMA are vulnerable to an array of morbidities and multi-morbidity across several physiologic systems and mental health disorders. Morbidity onset may occur earlier in the adult lifespan for those with vs. without SMA. Increasing clinical awareness of the high-early prevalence and OR of morbidities, improving screening strategies, and developing appropriate referral systems may help to reduce the burden of morbidities for this vulnerable population.

### Electronic supplementary material

Below is the link to the electronic supplementary material.

Supplementary Material 1

Supplementary Material 2

## Data Availability

The data that support the findings of this study are available from the Centers for Medicare & Medicaid Services (https://www.cms.gov) and Optum’s de-identified Clinformatics® Data Mart Database (https://www.optum.com/life-sciences-solutions.html) but restrictions apply to the availability of these data, which were used under license for the current study, and so are not publicly available.
