# Supplementary material for: Prevalence of morbidities across the lifespan for adults with spinal muscular atrophy: a retrospective cohort study
Source: Orphanet J Rare Dis. 2023 Aug 31;18:258. doi: 10.1186/s13023-023-02872-6 (PMC10472659; doi:10.1186/s13023-023-02872-6)
Supplement: Supplementary file 1 — Supplementary Material 1 [file 13023_2023_2872_MOESM1_ESM.docx]

**Additional file 1.** International Classification of Diseases, Ninth (ICD-9) and Tenth (ICD-10) Revision, Clinical Modification codes to identify variables for this study.

|  | **ICD-9 codes** | **ICD-10 codes** |
| --- | --- | --- |
| **Cohort** | | |
| Spinal muscular atrophy | 335.0, 335.1x | G12.0, G12.1, G12.8, G12.9 |
| **Morbidities** | | |
| Hypertension | 401.1, 401.9, 402.10, 402.90, 404.10, 404.90, 405.1x | I10.x-I13.x, I15.x |
| Epilepsy | 345.x | G40.x |
| Chronic pulmonary disease | 416.8, 416.9, 490.x-505.x, 506.4, 508.1, 508.8, 518.83 | I27.8, I27.9, J40.x-J47.x, J60.x-J67.x, J68.4, J70.1, J70.3, J96.10 |
| Depression | 300.4, 301.12, 309.0, 309.1, 311 | F20.4, F31.3-F31.5, F32.x, F33.x, F34.1, F41.2, F43.2 |
| Anemia, including blood loss and deficiency anemias | 280.x-281.9, 285.9 | D50.0, D50.8, D50.9, D51.x-D53.x |
| Gastrointestinal issues | 531.70, 531.90, 532.70, 532.90, 533.70, 533.90, 534.70, 534.90, 556.x, 564.0x, 564.1, V12.71 | K25.7, K25.9, K26.7, K26.9, K27.7, K27.9, K28.7, K28.9, K50.x-K52.x, K59.0, K58.x |
| Osteoarthritis and allied disorders | 715.x | M15.x-M19.x |
| Intellectual disabilities | 270.1, 317.x-319.x, 758.0, 758.1, 759.81, 759.83, | F70.x-F73.x, F78.x, F79.x, Q87.11, Q90.x, Q91.x, Q99.2 |
| Fluid and electrolyte disorders | 276.x | E22.2, E86.x, E87.x |
| Cardiac arrhythmias | 426.10, 426.11, 426.13, 426.2-426.53, 426.6-426.89, 427.0, 427.2, 427.31, 427.60, 427.9, 785.0, V45.0, V53.3 | I44.1-I44.3, I45.6, I45.9, I47.x-I49.x, R00.0, R00.1, R00.8, T82.1, Z45.0, Z95.0 |
| Hypothyroidism | 243-244.2, 244.8, 244.9 | E00.x-E03.x, E89.0 |
| Cerebrovascular disease | 362.34, 430.x-438.x | G45.x, G46.x, H34.0, I60.x-I69.x |
| Pneumonia | 480.x-486.x | J13, J14, J15.x, J16.x, J17, J18.x |
| Diabetes | 249.x, 250.x | E08.x-E13.x |
| Renal disease | 403.01, 403.11, 403.91, 404.02, 404.03, 404.12, 404.13, 404.92, 404.93, 582.x, 583.0-583.7, 585.x, 586.x, 588.0, V42.0, V45.1, V56.x | I12.0, I13.1, N03.2-N03.7, N05.2-N05.7, N18.x, N19.x, N25.0, Z49.0-Z49.2, Z94.0, Z99.2 |
| Cancer, includes: (1) any malignancy, including lymphoma and leukemia, except malignant neoplasm of skin; and (2) metastatic cancer | 140.x-172.x, 174.x-195.8, 196.x-199.1, 200.x-208.x, 238.6, V10.00-V10.9 | C00.x-C26.x, C30.x-C34.x, C37.x-C41.x, C43.x, C45.x-C58.x, C60.x-C76.x, C77.x-C80.x, C81.x-C85.x, C88.x, C90.x-C97.x |
| Congestive heart failure | 398.91, 402.11, 402.91, 404.11, 404.13, 404.91, 404.93, 428.x | I09.9, I11.0, I13.0, I13.2, I25.5, I42.0, I42.5-I42.9, I43.x, I50.x |
| Bone fragility | 733.0x, 733.1x, 733.8x, 733.93-733.98, 805.x-829.x | M80.x, M81.x, M84.3-M84.9, S12.x, S22.x, S32.x, S42.x, S52.x, S62.x, S72.x, S82.x, S92.x |
| Neurogenic bowel or bladder | 564.81, 596.54 | K59.2, N31.x |
| Liver disease | 070.22, 070.23, 070.32, 070.33, 070.44, 070.54, 070.6, 070.9, 456.0-456.2, 570.x, 571.x, 572.2-572.8, 573.3, 573.4, 573.8, 573.9, V42.7 | B18.x, K70.0-K70.3, K70.9, K71.3-K71.5, K71.7, K73.x, K74.x, K76.0, K76.2-K76.4, K76.8, K76.9, Z94.4, I85.0, I85.9, I86.4, I98.2, K70.4, K71.1, K72.1, K72.9, K76.5, K76.6, K76.7 |
| Dementia | 290.x, 294.1, 331.0, 331.2 | F00.x-F03.x, F05.1, G30.x, G31.1 |
| Myocardial infarction | 410.x, 412.x | I21.x, I22.x, I25.2 |
| Rheumatoid arthritis and other inflammatory polyarthropathies | 714.x | M05.x, M06.x, M08.x |
| Anxiety | 300.0x | F40.x-F45.x, F48.x |
| Sleep disorders, without hypoventilation | 307.4x, 327.0, 327.1, 327.20-327.23, 327.27, 327.28, 327.3-327.5, 327.8, 347.x, 780.5x | F51.x, G47.0x-G47.2x, G47.30-G47.33, G47.37, G47.39 |
| Sleep related hypoventilation | 327.24-327.26 | G47.34-G47.36 |
| Psychoses | 295.x-298.x | F20.x, F22.x-F25.x, F28.x, F29.x, F30.2, F31.2, F31.5 |
| Urine incontinence | 788.3 | N39.3, N39.4x, R32.x |
| Migraine | 346.x | G43.x |
| Alcohol or drug abuse | 291.x, 292.x, 303.90-303.93, 304.x, 305.0x, 305.20-305.93, V11.3 | F10.x-F16.x, F18.x, F19.x, G62.1, I42.6, K29.2x, K70.x, Z71.41, Z71.51x |
