# Supplementary material for: Prevalence of morbidities across the lifespan for adults with spinal muscular atrophy: a retrospective cohort study
Source: Orphanet J Rare Dis. 2023 Aug 31;18:258. doi: 10.1186/s13023-023-02872-6 (PMC10472659; doi:10.1186/s13023-023-02872-6)
Supplement: Supplementary file 2 — Supplementary Material 2 [file 13023_2023_2872_MOESM2_ESM.docx]

**Additional file 2.** Odds ratio (OR) of morbidities with effect modification by age, comparing adults with spinal muscular atrophy (n=2,427) to matched adults without spinal muscular atrophy (n=484,528).

|  | **18-39 years** | **40-54 years** | **55-64 years** | **65-74 years** | **≥75 years** |
| --- | --- | --- | --- | --- | --- |
|  | OR (95% CI) | OR (95% CI) | OR (95% CI) | OR (95% CI) | OR (95% CI) |
| Intellectual disabilities | 38.5 (22.0, 67.4) | 39.7 (24.1, 65.5) | * | * | * |
| Epilepsy | 10.2 (6.31, 16.6) | 11.2 (7.9, 16.0) | 6.17 (4.03, 9.45) | 4.13 (2.76, 6.18) | 4.76 (3.21, 7.04) |
| Hypertension | 4.47 (3.47, 5.75) | 2.73 (2.28, 3.28) | 2.54 (2.06, 3.12) | 1.97 (1.64, 2.35) | 2.08 (1.60, 2.71) |
| Congestive heart failure | 26.6 (14.4, 48.9) | 15.1 (11.1, 20.4) | 5.18 (3.90, 6.87) | 4.15 (3.42, 5.05) | 2.84 (2.34, 3.43) |
| Cardiac arrhythmias | 8.14 (6.29, 10.5) | 5.19 (4.16, 6.47) | 3.77 (3.05, 4.65) | 3.27 (2.80, 3.82) | 2.05 (1.72, 2.46) |
| Myocardial infarction | 15.5 (5.58, 43.2) | 6.35 (4.07, 9.91) | 3.99 (2.81, 5.67) | 2.64 (2.02, 3.45) | 1.90 (1.41, 2.55) |
| Cerebrovascular disease | 12.2 (6.75, 22.0) | 11.9 (9.04, 15.6) | 6.04 (4.75, 7.67) | 4.66 (3.94, 5.52) | 2.96 (2.46, 3.56) |
| Diabetes | 3.89 (2.64, 5.74) | 3.29 (2.66, 4.06) | 2.02 (1.65, 2.47) | 1.52 (1.29, 1.78) | 1.34 (1.11, 1.62) |
| Chronic pulmonary disease | 9.84 (7.83, 12.4) | 6.25 (5.11, 7.65) | 4.85 (3.98, 5.91) | 2.74 (2.34, 3.22) | 1.86 (1.54, 2.25) |
| Pneumonia | 38.7 (29.2, 51.1) | 19.1 (15.1, 24.2) | 9.34 (7.27, 12.0) | 5.99 (4.91, 7.30) | 4.35 (3.56, 5.31) |
| Liver disease | 5.57 (3.24, 9.56) | 3.55 (2.47, 5.10) | 3.30 (2.40, 4.53) | 2.08 (1.49, 2.90) | 2.16 (1.38, 3.38) |
| Renal disease | 21.1 (12.7, 35.1) | 6.77 (4.77, 9.61) | 4.04 (3.06, 5.35) | 2.70 (2.23, 3.28) | 1.93 (1.59, 2.36) |
| Hypothyroidism | 2.38 (1.62, 3.49) | 2.26 (1.75, 2.92) | 1.20 (0.90, 1.59) | 1.68 (1.39, 2.02) | 1.45 (1.18, 1.79) |
| Cancer | 3.84 (2.15, 6.86) | 2.13 (1.53, 2.98) | 1.68 (1.29, 2.18) | 1.84 (1.55, 2.19) | 1.30 (1.06, 1.59) |
| Bone fragility | 6.76 (4.96, 9.21) | 7.83 (6.19, 9.92) | 4.85 (3.85, 6.12) | 3.43 (2.87, 4.09) | 2.97 (2.46, 3.60) |
| Osteoarthritis | 8.30 (5.35, 12.9) | 4.47 (3.54, 5.63) | 3.35 (2.73, 4.11) | 2.48 (2.11, 2.91) | 2.66 (2.22, 3.19) |
| Dementia | * | 59.2 (33.1, 106.1) | 16.8 (10.7, 26.6) | 7.84 (6.06, 10.2) | 3.35 (2.75, 4.09) |
| Sleep disorders (excludes sleep-related hypoventilation) | 7.02 (5.42, 9.09) | 3.61 (2.92, 4.46) | 3.00 (2.42, 3.72) | 2.50 (2.08, 2.99) | 2.55 (2.02, 3.21) |
| Sleep-related hypoventilation | * | * | * | * | * |
| Neurogenic bladder or bowel | 117.7 (66.5, 208.4) | 62.2 (42.4, 91.3) | 36.8 (24.8, 54.7) | 21.2 (15.2, 29.6) | 5.96 (3.54, 10.0) |
| Anemia | 7.93 (5.97, 10.5) | 7.50 (6.06, 9.27) | 5.90 (4.78, 7.28) | 4.40 (3.74, 5.16) | 2.82 (2.35, 3.38) |
| Fluid/electrolyte disorders | 24.8 (18.9, 32.4) | 15.8 (12.8, 19.5) | 8.59 (6.94, 10.6) | 5.89 (4.99, 6.96) | 4.90 (4.09, 5.88) |
| Gastrointestinal issues | 8.07 (6.03, 10.8) | 9.29 (7.43, 11.6) | 6.36 (5.02, 8.06) | 4.92 (4.08, 5.92) | 4.19 (3.43, 5.10) |
| Urine incontinence | 39.8 (26.4, 60.0) | 17.4 (12.7, 23.9) | 11.5 (8.51, 15.5) | 4.37 (3.36, 5.69) | 3.17 (2.43, 4.13) |

CI, confidence interval. *Too few outcome events to provide a meaningful estimate.
